# Laparoscopic Cholecystectomy in a Patient with Previous Pneumonectomy: A Case Report and Discussion of Anaesthetic Considerations

**DOI:** 10.1155/2014/582078

**Published:** 2014-11-09

**Authors:** Dash Faith Newington, Sanaa Ismail

**Affiliations:** ^1^Department of Anaesthesia, Launceston General Hospital, Charles Street, Launceston, TAS 7250, Australia; ^2^Department of Anaesthesia, Dubbo Base Hospital, Myall Street, Dubbo, NSW 2830, Australia

## Abstract

Increasing numbers of patients require cholecystectomy after previous pneumonectomy, but there are little data to guide anaesthetic management. A laparoscopic approach is associated with less postoperative respiratory compromise than open cholecystectomy but may be relatively contraindicated due to the undesirable effects of pneumoperitoneum on respiratory function. We describe the case of a 72-year-old patient who successfully underwent elective laparoscopic cholecystectomy 23 years after left pneumonectomy. An understanding of the combined physiological consequences of pneumonectomy and pneumoperitoneum facilitated the provision of safe and uneventful anaesthesia. We propose that laparoscopic cholecystectomy is feasible and safe to perform in patients with a single lung.

## 1. Introduction

Pulmonary disease is a well-established risk factor for perioperative respiratory complications. Pneumonectomy represents an extreme example of pulmonary compromise induced by a therapeutic surgical procedure. However, with five-year survival rates in patients undergoing pneumonectomy exceeding 40% for malignant disease [1–3] and 75% for benign disease [4–6], anaesthetists can reasonably expect to encounter an increasing number of these patients presenting for elective or emergency surgery. Most surgical experience after pneumonectomy has involved cardiac procedures and further resections of the remaining lung. Published data to guide anaesthetic decision-making in patients undergoing cholecystectomy for laparoscopic procedures after pneumonectomy are scarce. A laparoscopic approach is ordinarily associated with less postoperative pulmonary complications than open cholecystectomy [7] but has not been specifically studied in patients with previous lung resection. Here we present the case of an elderly man who underwent laparoscopic cholecystectomy 23 years after left pneumonectomy in order to illustrate the salient anaesthetic considerations of this increasingly common scenario.

## 2. Case Presentation

A 72-year-old 80 kg man with a history of recurrent biliary pancreatitis presented to our hospital for elective laparoscopic cholecystectomy after recently being declined the procedure at a nearby tertiary centre. The patient had undergone left standard pneumonectomy with mediastinal lymph node resection for Stage 1 (T2N0M0) bronchogenic squamous cell carcinoma as a curative procedure 23 years earlier, without adjuvant or neoadjuvant chemo- or radiotherapy. He had no recurrent malignant disease. The patient ceased smoking at the time of his cancer diagnosis but had accrued a 30-pack-year history with consequent emphysematous lung disease on a background of asthma (treated with regular inhaled salbutamol, tiotropium bromide, and combination of salmeterol/fluticasone). His chronic airways disease was optimally managed upon presentation for cholecystectomy, although he had required hospitalisation for a life-threatening infective exacerbation six months previously.

The patient's medical history was also relevant to stable angina, a history of a transient ischaemic attack, peripheral vascular disease with recent revascularisation, polymyalgia rheumatica treated with long-term systemic corticosteroids (10 mg of prednisone daily over many years), chronic pain secondary to multiple vertebral crush fractures, and stage three renal impairment. He described mild exertional dyspnea with an exercise tolerance of two flights of stairs. Our patient had not been subject to general anaesthesia since his pneumonectomy, although had undergone multiple procedures under sedation, which were tolerated well.

**Figure 1:**
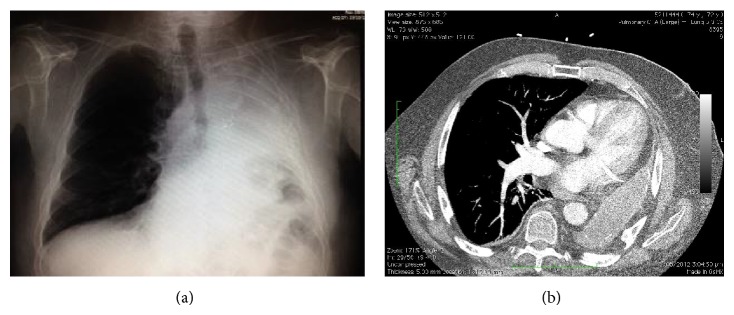
Preoperative chest X-ray and CT scan.

On examination, our patient had a respiratory rate of 16 and a baseline SpO_2_ of 93%. Auscultation of the chest revealed good air entry on the right with no adventitious sounds. No breath sounds were audible on the left. Heart sounds were faint but dual and the ECG showed sinus rhythm with inferior *Q* waves. Preoperative (along with intraoperative) arterial blood gas findings are shown in Table 1. Haemoglobin and haematocrit were within normal limits. Spirometry demonstrated an FVC of 1.6L (33% of predicted), FEV1 of 1.05L (30% of predicted), and FEV1/FVC of 65%. Images of the preoperative chest X-ray and recent CT scan of the chest performed for another indication are shown in Figure 1. A transthoracic echocardiogram suggested mild pulmonary hypertension with largely preserved right and left heart function. A coronary angiogram performed 18 months previously for atypical chest pain revealed a chronically occluded right coronary artery not suitable for percutaneous intervention, mild left coronary disease, and minor irregularities of the circumflex. On ventriculography, left ventricular systolic contraction was mildly impaired with localised inferior hypokinesis consistent with a previous infarct.

In elective settings where patients face high perioperative risk in the context of unclear functional reserve, cardiopulmonary exercise testing can quantify exercise limitation and determine whether any deficit is primarily respiratory or cardiovascular in nature [8]. Limitations of cost and availability precluded its use in our patient.

Note the deviation of heart and mediastinal structures into the left hemithorax, elevation of the left hemidiaphragm, and compensatory emphysema of the right lung. The CT scan also revealed large apical bullae in the remaining lung and elevation of the spleen and left kidney toward the left hemithorax, which are not visible on this slice.

After preoxygenation, general anaesthesia was induced and maintained using a total intravenous technique with propofol and remifentanil target controlled infusions. Intravenous ketamine was also used on induction for its bronchodilating effect and as an opioid-sparing analgesic. After administration of cisatracurium, hydrocortisone (100 mg, in addition to his usual 10 mg of prednisone on the morning of surgery), and prophylactic antibiotics, elective use of the McGrath video-laryngoscope revealed a grade 1 view of the larynx. A size 8.0 cuffed oral Magill endotracheal tube was passed easily into the trachea without an introducer. No attempt was made at right endobronchial intubation. Mechanical ventilation was commenced using a pressure-controlled mode with volume guarantee (450 mL × 14/min). Peak, plateau, and positive end expiratory pressures were 23, 8, and 0 cmH20, respectively.

An arterial cannula was inserted to facilitate repeat arterial gas sampling, as etCO_2_ was considered unlikely to accurately reflect PaCO_2_ in this context. Carbon dioxide pneumoperitoneum was maintained at 10–12 mmHg. 1 mg of metaraminol, in divided doses, maintained satisfactory mean arterial pressure throughout the 70-minute procedure. The patient was weaned to pressure support ventilation 15 minutes prior to an uneventful extubation to CPAP in the reverse Trendelenburg position. After extubation in the operating theatre, the patient was admitted to the high dependency unit for postoperative monitoring. The patient received 25 mg of intravenous hydrocortisone, six-hourly, until being able to resume his regular oral prednisone. The following morning he was transferred to the general surgical ward with no requirement for supplemental oxygen. He was discharged home on the second postoperative day.

## 3. Discussion

Here we present the third reported case of a patient undergoing cholecystectomy after previous pneumonectomy. We were unable to find any case report in which an open surgical approach was used. We found a single case report describing a laparoscopic procedure other than cholecystectomy, in which an uneventful Toupet fundoplication and anterior gastropexy were performed for repair of a gastric volvulus occurring 33 years after left pneumonectomy [9]. The two previously reported cases of laparoscopic cholecystectomy involved emergency procedures in elderly female patients, 30 years after left [10] and nine years after right pneumonectomy [11]. In the first case, endotracheal general anaesthesia was employed using volume-controlled ventilation (tidal volume of 5 mL/kg producing airway pressures of 15–20 cmH20). Abdominal insufflation pressures were not reported. The second case was performed under combined spinal epidural anaesthesia with midazolam sedation. Whilst receiving supplemental oxygen via a Hudson mask, the patient maintained spontaneous ventilation throughout the 45-minute procedure, which was performed at an insufflation pressure of 9 mmHg. In both cases, the patient had an uneventful perioperative course and made a full recovery.

The postpneumonectomy state is associated with predictable anatomic and physiological changes [12]. With time, the heart and mediastinum deviate toward the side of the resected lung, by counterclockwise rotation after left pneumonectomy and by translocation after right pneumonectomy. The remaining lung develops compensatory hyperinflation and frequently herniates across the midline. Elevation of the ipsilateral hemidiaphragm shifts the liver or spleen in a cephalad direction. Mild thoracic scoliosis is common secondary to changes in the shape of the thoracic cage [13].

Right pneumonectomy is associated with a threefold greater mortality than left pneumonectomy [14, 15]. Reasons for this are unclear, although anatomical factors predispose to a higher incidence of serious early and late complications after right-sided surgery. These include bronchopleural fistula and empyema, postpneumonectomy pulmonary oedema, postoperative arrhythmias, pulmonary artery thrombosis, and the postpneumonectomy syndrome in which long-term anatomical changes result in stretching and extrinsic compression of the tracheobronchial tree and oesophagus [12].

Respiratory reserve is diminished after pneumonectomy, but to a lesser extent than would be expected from the size of the resection. Pulmonary function tests characteristically reveal a mixed restrictive and obstructive pattern with a decrease in lung compliance and an increase in airway resistance [12]. FVC and FEV1 are decreased to a greater extent than exercise capacity [16]. There is minimal change in subjective sensation of breathlessness as measured by Borg scores [17]. Postprocedural PaO_2_ and PaCO_2_ are proportional to the preoperative health of the remaining lung [12].

Although a laparoscopic approach is now the gold-standard surgery for cholecystectomy [18], the associated pneumoperitoneum presents a physiological challenge to patients with limited respiratory reserve. The raised intra-abdominal pressure compresses the lung, reducing diaphragmatic excursion, compliance, and functional residual capacity. Higher plateau and peak airway pressures are required to achieve a given tidal volume. These effects are pressure dependent, such that greater derangements occur with higher intra-abdominal pressures. The key principle of laparoscopic surgery to use the minimum insufflation pressure compatible with acceptable surgical conditions is of particular importance in patients with impaired respiratory function [19].

Carbon dioxide is the agent of choice for pneumoperitoneum because it is widely available, is inexpensive, does not support combustion, is readily absorbed, and poses less risk of gas embolism than other agents. However, significant transperitoneal absorption of CO_2_ is inevitable. Allowing a patient to breathe spontaneously risks hypercarbia and acidosis. With controlled ventilation, minute volume can be adjusted to counterbalance this effect. We therefore chose controlled ventilation for our patient. Although mechanical ventilation is a risk factor for early bronchopleural fistulae after pneumonectomy, it does not increase the risk of late bronchopleural fistula after the first 90 postoperative days [20].

An endotracheal tube was our airway of choice to facilitate controlled ventilation. With an increase in intraabdominal pressure, there is a risk that an endotracheal tube may advance and become endobronchial. Care should be taken to avoid inadvertent endobronchial intubation on the same side as a previous pneumonectomy.

It was necessary to hyperventilate our patient to achieve a neutral acid-base balance. We chose to increase rate rather than tidal volume as we felt that the risk of volutrauma and pneumothorax in the lone lung outweighed the risk of gas trapping. Upon release of the pneumoperitoneum we applied extrinsic PEEP and employed recruitment maneuvers to minimise alveolar dead space.

Functional changes in oesophageal motility are common in patients with a history of pneumonectomy [21, 22], although most patients do not report dysphagia [13]. It is unclear whether these patients are at increased risk of aspiration during anaesthesia. However it seems prudent to take all reasonable measures to protect the remaining lung from such an insult.

In conclusion, an understanding of the physiological consequences of both pneumonectomy and pneumoperitoneum allowed us to implement measures to preserve the function of the single remaining lung and safely provide anaesthesia for laparoscopic cholecystectomy in a patient who had previously undergone pneumonectomy. Adding to the two previously reported cases, both with successful outcomes, our experience suggests that laparoscopic cholecystectomy may be feasible and safe to perform after previous pneumonectomy. Laparoscopic procedures other than cholecystectomy may also be practicable in such patients, although there is not yet enough experience to guide management.

## Figures and Tables

**Table 1:** Arterial blood gas results.

	Preoperative	Intraoperative
FiO_2_	0.21	0.6
pH	7.32	7.31
PaO_2_ (mmHg)	98	152
PaCO_2_ (mmHg)	60	53
HCO_3_	30	26
BE	3	−1
